# Machine learning and radiomics for segmentation and classification of adnexal masses on ultrasound

**DOI:** 10.1038/s41698-024-00527-8

**Published:** 2024-02-20

**Authors:** Jennifer F. Barcroft, Kristofer Linton-Reid, Chiara Landolfo, Maya Al-Memar, Nina Parker, Chris Kyriacou, Maria Munaretto, Martina Fantauzzi, Nina Cooper, Joseph Yazbek, Nishat Bharwani, Sa Ra Lee, Ju Hee Kim, Dirk Timmerman, Joram Posma, Luca Savelli, Srdjan Saso, Eric O. Aboagye, Tom Bourne

**Affiliations:** 1 Department of Metabolism, Digestion and Reproduction, Imperial College London, London, UK; 2 Department of Obstetrics and Gynaecology, Imperial College Healthcare NHS Trust, London, UK; 3 Department of Surgery and Cancer, Imperial College London, London, UK; 4 Department of Obstetrics and Gynaecology, Ospedale Morgagni-Pierantoni, Forli, Italy; 5 Department of Medicine and Surgery, University of Milan-Bicocca, Milan, Italy; 6 Department of Radiology, Imperial College Healthcare NHS Trust, London, UK; 7 Department of Obstetrics and Gynaecology, Asan Medical Center, Seoul, South Korea; 8 Department of Obstetrics and Gynecology, University Hospitals Leuven, Leuven, Belgium; 9 Department of Development and Regeneration, KU Leuven, Leuven, Belgium

**Keywords:** Cancer imaging, Cancer imaging

## Abstract

Ultrasound-based models exist to support the classification of adnexal masses but are subjective and rely upon ultrasound expertise. We aimed to develop an end-to-end machine learning (ML) model capable of automating the classification of adnexal masses. In this retrospective study, transvaginal ultrasound scan images with linked diagnoses (ultrasound subjective assessment or histology) were extracted and segmented from Imperial College Healthcare, UK (ICH development dataset; *n* = 577 masses; 1444 images) and Morgagni-Pierantoni Hospital, Italy (MPH external dataset; *n* = 184 masses; 476 images). A segmentation and classification model was developed using convolutional neural networks and traditional radiomics features. Dice surface coefficient (DICE) was used to measure segmentation performance and area under the ROC curve (AUC), F1-score and recall for classification performance. The ICH and MPH datasets had a median age of 45 (IQR 35–60) and 48 (IQR 38–57) years old and consisted of 23.1% and 31.5% malignant cases, respectively. The best segmentation model achieved a DICE score of 0.85 ± 0.01, 0.88 ± 0.01 and 0.85 ± 0.01 in the ICH training, ICH validation and MPH test sets. The best classification model achieved a recall of 1.00 and F1-score of 0.88 (AUC:0.93), 0.94 (AUC:0.89) and 0.83 (AUC:0.90) in the ICH training, ICH validation and MPH test sets, respectively. We have developed an end-to-end radiomics-based model capable of adnexal mass segmentation and classification, with a comparable predictive performance (AUC 0.90) to the published performance of expert subjective assessment (gold standard), and current risk models. Further prospective evaluation of the classification performance of this ML model against existing methods is required.

## Introduction

Ovarian Cancer (OC) affects 2% of women in their lifetime and remains the leading cause of death from a gynaecological malignancy in the UK^1^. The poor prognosis of OC is mainly attributed to most women (75%) presenting late, with advanced stage disease^1^. Unfortunately, an effective OC screening programme does not exist; therefore, diagnosis is reliant upon prompt recognition of gynaecological symptoms and accurate interpretation of clinical imaging^2,3^.

Adnexal masses are common, affecting up to 18% of postmenopausal women in the UK^4^. The accurate classification of adnexal masses is fundamental to ensure malignant adnexal masses are promptly identified and undergo surgical intervention by an appropriately trained surgeon. Particularly in younger women and those with an asymptomatic lesion, it is important that those with a benign mass are not subjected to unnecessary intervention, with potential complications^5^.

Expert subjective assessment (SA) is the gold standard method for classifying adnexal masses, yet is restricted by the availability of expert examiners^6^. Various ultrasound-based diagnostic models, using a combination of ultrasound features with or without serological markers (CA-125) exist to support the classification of adnexal masses, including the Risk of malignancy index (RMI)^7^, International Ovarian Tumour Analysis (IOTA) Simple Rules (SR)^8^, the IOTA Assessment of Different NEoplasia’s in the adneXa (ADNEX) model^9^ and American College of Radiology (ACR) Ovarian-Adnexal Reporting and Data System Ultrasound (ORADS-US)^10^.

ADNEX (with CA-125) is the best-performing ultrasound-based model with an AUC of 0.94, sensitivity of 0.865 (fixed specificity 0.90) and specificity of 0.866 (fixed sensitivity 0.90)^9,11^. RMI is the recommended approach within UK National Guidance for the assessment of adnexal masses in post-menopausal women^7^. RMI has an AUC of 0.89, sensitivity of 0.701 (fixed specificity 0.90) and specificity of 0.693 (fixed sensitivity 0.90)^12^. However, expert SA remains the best method for classifying adnexal masses, with an AUC of 0.96, sensitivity of 0.90 and specificity of 0.91^6^. Although ultrasound-based models have been internally and externally validated in the hands of expert and non-expert ultrasound examiners, their use involves the subjective interpretation of ultrasound-based features^6,11,13^.

Machine learning (ML) refers to the enablement of computers to perform tasks without the need for explicit programming. Deep Learning (DL) is a subset of ML, that specifically refers to the use of artificial neural networks, which has shown great promise within clinical image analysis^14^. Typically, DL models consist of a series of interconnecting neural networks and nodes, which form connections through a process of supervised training, involving repeated exposure to adnexal mass images, linked to the clinical/histological diagnosis^14^. Radiomics broadly defines a process which involves extracting high-throughput quantitative features from images^15^. Radiomics includes first-order statistics such as the median and mean pixel intensity and higher-order features such as textural features, and functions of fractal/wavelet transformed images^15^.

Given the differences in the morphology between benign and malignant adnexal masses on ultrasound, we would anticipate a similar discrete pattern in the radiomics features (signature) which could be utilised to develop an ML classification model. A radiomics-based model provides a degree of mathematical explainability to the classification output, which is invaluable within clinical image interpretation^16^.

Current methods of adnexal mass classification rely on ultrasound experience and are prone to error^13^. There is a need for a robust ML model, capable of classifying adnexal masses, which does not rely upon prior ultrasound experience, and can provide a scalable, generalisable, accurate solution to adnexal mass classification.

There has been recent interest in the integration of ML into the ultrasound classification of adnexal masses^17^. A recent meta-analysis and systematic review highlighted 17 studies applying ML models (principally DL) in this field^17–26^. The pooled model performance had a sensitivity of 0.91 and specificity of 0.87^17^. The most recent studies by Gao et al. and Li et al., published in 2022, both used a DL-based approach to automate the classification of adnexal masses and evaluated the performance of the models in an external validation test set^19,27^. All previous studies using an end-to-end ML-based approach to classify adnexal masses, have not evaluated the performance on an external validation data set^18,21,25,28^. In the Gao et al. paper, the DL model had an AUC of 0.911 and an F1-score of 0.812 in the internal validation, with a noticeable drop in performance in the external test set, with an AUC of 0.870, but an F1-score of 0.551 and a recall (sensitivity) of 0.403^19^. The Li et al. group, developed a DL-based model to automate the classification of benign, borderline and malignant masses using both transabdominal and transvaginal images, with an F1 score of 0.746 and 0.684 and a recall (sensitivity) of 0.907 and 0.800 in test set 1 and 2, respectively^27^. The F1-score adjusts for class imbalance (true negatives do not contribute to the score) and so is the performance metric of choice when evaluating an ML model, rather than AUC.

Our study aims to extend our previous work in Computed Tomography (CT) scanning, which focused on the development and validation of a radiomics-based model to improve the prediction of the prognosis for women with OC^29,30^. We aim to develop and externally validate a robust ML model, utilising both radiomics and DL approaches, capable of classifying adnexal masses on ultrasound. In addition, we shall determine the value of integrating various clinical parameters such as CA-125 and age on the classification performance of the model. Finally, recognising the importance of a robust, generalisable model its performance will be evaluated on an external test set of adnexal masses.

## Results

### Clinical characteristics of data

The ICH development dataset consisted of 577 cases (1444 images); the median age was 45 years old (IQR 35–60) (Table 1). All malignant cases (23.1%) were managed surgically and high-grade serous carcinoma (*n* = 41, 7.1%) was the commonest malignant adnexal mass. Most benign cases were managed conservatively (*n* = 292, 65.8%) and cystadenoma (*n* = 179, 31.0%) was the commonest benign mass (Table 2). Serum CA-125 levels were available for 301 cases (52.2%), with a median value of 25 U/ml (IQR 14–114) (Table 1).

**Table 1:** Summary of the characteristics of the ICH Development (training and internal validation) and MPH External test set: patient demographics, presence of histological diagnosis and CA-125 level

Parameter		ICH Development *n* = 577, 1444 images	MPH Test *n* = 184, 476 images	*P* value
Age: median (IQR) years		45 (35–60)	48 (38–57)	0.63
Number of images (median, IQR)		2 (2–3)	2 (2–3)	
Diagnosis	Benign	444 (76.9%)	126 (68.5%)	0.029
	Malignant	133 (23.1%)	58 (31.5%)	
Histology (%)	Yes	285 (49.4%)	184 (100.0%)	2.22 × 10^−16^
	No	292 (50.6%)		
CA-125 U/ML (median, IQR)		25 (14–114)	16.5 (10–54.8)	0.062

The differences between individual parameters are demonstrated with the respective *p* values.

**Table 2:** Adnexal mass diagnosis, based on histology or ultrasound (expert subjective assessment) within the ICH development (training and internal validation) and MPH external test set (*n*, %)

Type of adnexal mass	*N*, % ICH Development (training and validation)	*N,* % MPH Test Data
Cystadenoma (serous, mucinous, seromucinous)	179 (31.0%)	44 (23.9%)
Dermoid	100 (17.3%)	31 (16.9%)
Endometrioma	56 (9.7%)	33 (17.9%)
High-Grade Serous Carcinoma	41 (7.1%)	8 (4.3%)
Cystadenofibroma	40 (6.9%)	4 (2.2%)
Benign tubal	31 (5.4%)	0 (0.0%)
Serous Borderline	21 (3.7%)	15 (8.2%)
Mucinous Borderline	17 (3.0%)	1 (0.5%)
Fibroma	18 (3.1%)	8 (4.3%)
Benign Other	15 (2.6%)	5 (2.7%)
Endometroid ovarian cancer	10 (1.7%)	2 (1.1%)
Clear cell ovarian cancer	7 (1.2%)	2 (1.1%)
Sex cord	7 (1.2%)	10 (5.4%)
Struma Ovarii	5 (0.9%)	0 (0.0%)
Metastasis	5 (0.9%)	4 (2.2%)
Mucinous Carcinoma	4 (0.7%)	6 (3.3%)
Malignant other	4 (0.7%)	2 (1.1%)
Carcinosarcoma	6 (1.0%)	3 (1.6%)
Seromucinous Borderline	6 (1.0%)	6 (3.3%)
Germ cell	3 (0.5%)	0 (0.0%)
Other Borderline (Brenner, Endometroid)	2 (0.4%)	0 (0.0%)
TOTAL	577 (100.0%)	184 (100.0%)

There was a significant difference in adnexal mass sub-types between the two datasets (ICH, MPH), demonstrated with a *p* value of 0.000000027.

The MPH test dataset consisted of 184 cases (476 images); the median age was 48 years old (IQR 38–57). All MPH cases were managed surgically, with a malignancy rate of 31.5%, which was significantly higher than ICH (*p* = 0.029). CA-125 data were available for 108 cases (58.7%), with a median value of 16.5 U/ml (IQR 10–54.8). Serous borderline (*n* = 15, 8.2%) and cystadenoma (*n* = 44, 23.9%) were the most common malignant and benign adnexal masses respectively (Tables 1, 2). The difference in adnexal sub-types between the ICH and MPH dataset were statistically significant (*p* < 0.001). The MPH test data set contained 150 images (31.5%) with callipers present, we found that the presence of callipers did not contribute to the explained variance within the data set (Supplementary Fig. 4).

### Model performance

**Fig. 1:**
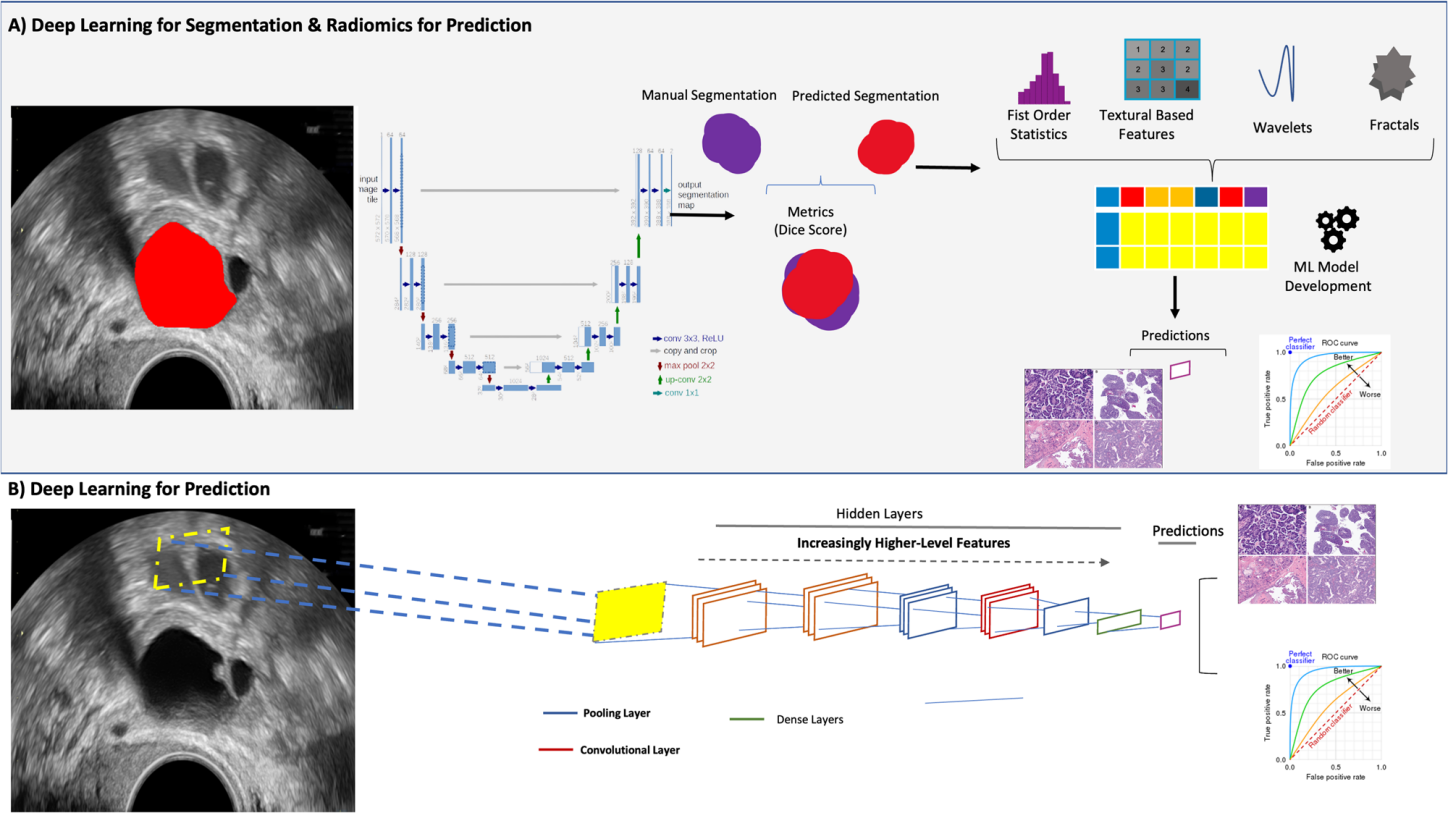
Overview of AI approach. An end-to-end approach can either rely on a combination of Deep Learning (DL) for segmentation and radiomics, or a direct DL-based approach without the need for segmentation. **A** The traditional radiomics feature pipeline from segmented region of interest to radiomics feature computation and Machine learning (ML) modelling. **B** DL approach, using convolutional neural networks (CNN), to facilitate auto-segmentation and classification.

**Fig. 2:**
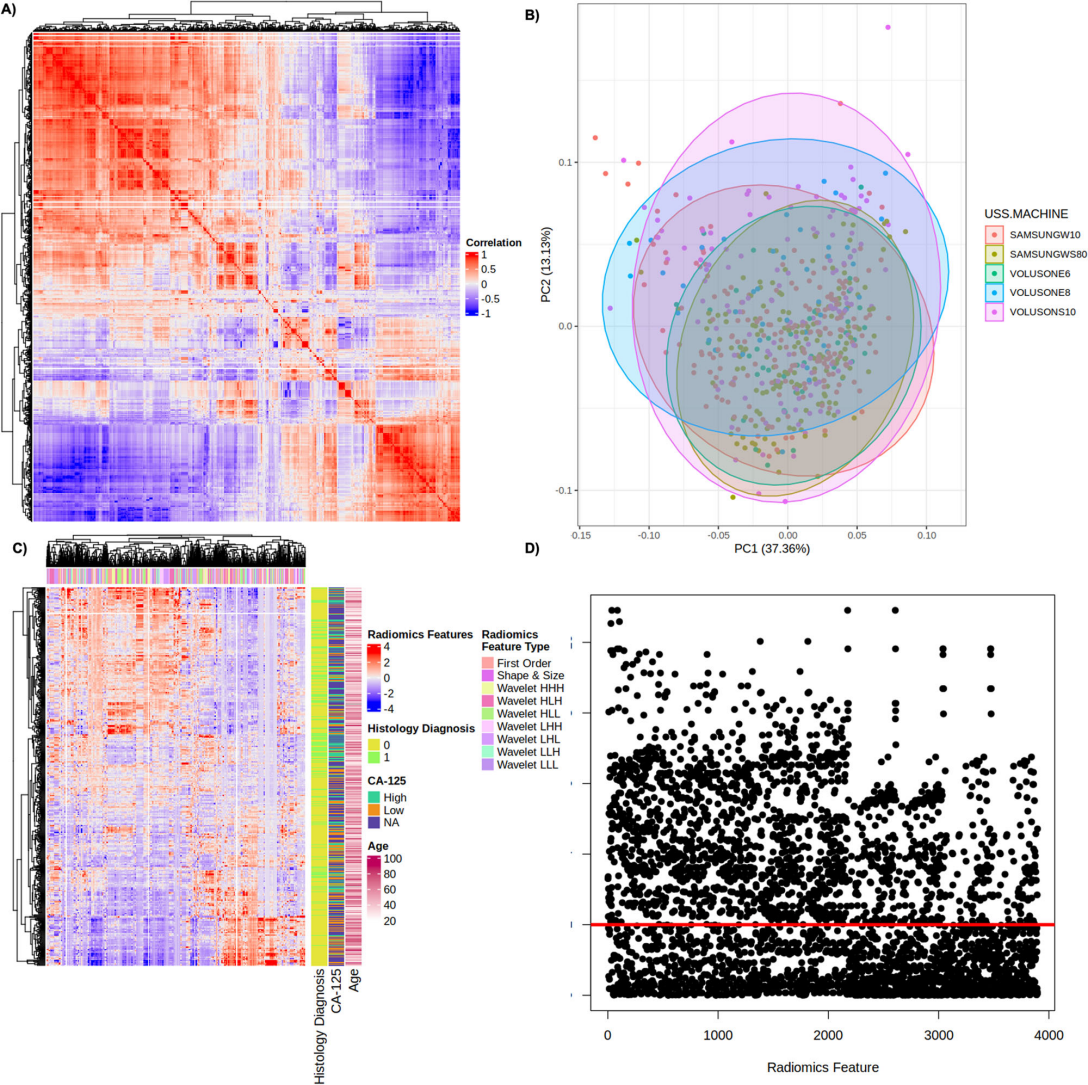
Overview of Radiomics data structure. **A** Correlation matrix heat map of radiomics parameters. The degree of correlation between radiomics parameters is indicated within the heatmap (red indicates perfect correlation). **B** Principal component analysis (PCA) indicates the degree of explained variance within the dataset (ultrasound scanner type), PC1 and PC2 explain 37.6% and 13.14% of explained variance. **C** Heatmap of all extracted radiomics features for adnexal mass classification. Each row corresponds to an individual patient and each column corresponds to each scaled radiomics feature. The colour key outlines the corresponding radiomics feature sub-type. Clinical parameters including age and CA-125 are represented. **D** Univariate logistic regression outlining radiomics features and their respective univariate logistic regression derived *p* values (horizontal red line indicates *p* < 0.01).

**Fig. 3:**
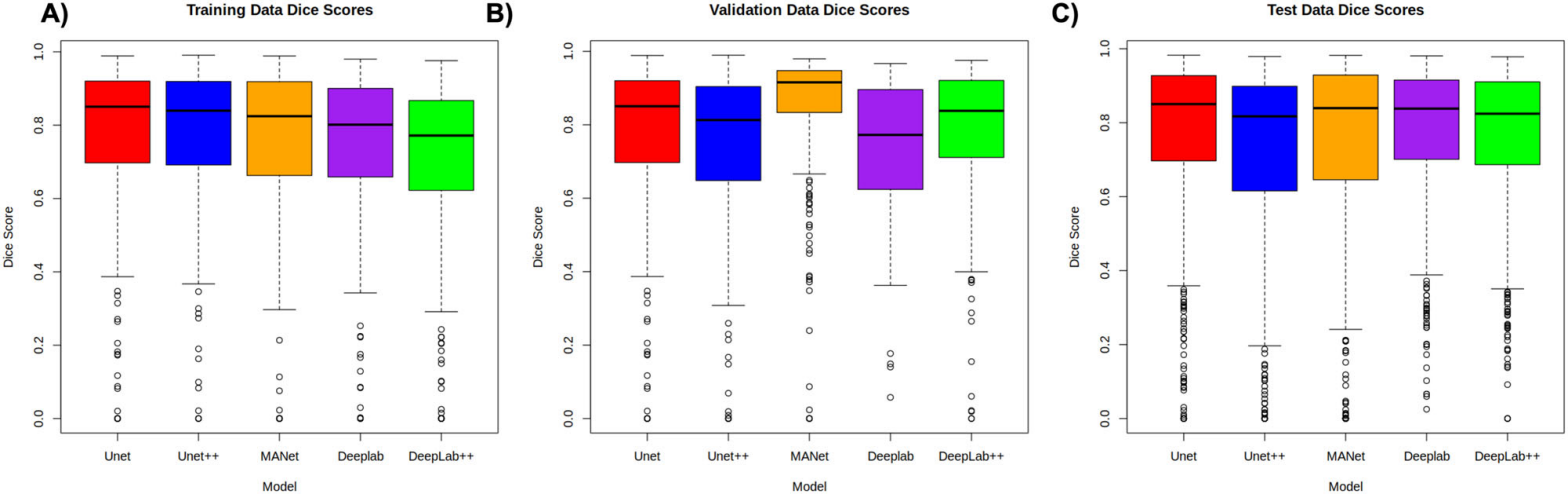
The performance of five Deep Learning (DL) segmentation models. (1) Unet (2) Unet++ (3) MANet (4) Deeplab (5) Deeplab++ compared to ground truth segmentation within (**A**) ICH training dataset, (**B**) ICH validation dataset, (**C**) MPH test dataset. The similarity scores (dice surface coefficient, Dice scores), presented within a box plot, the middle line corresponds to the median, the upper and lower boundaries of the box correspond to upper and lower quartiles, whilst the whiskers reflect the minimum and maximum value and the white dots below the whiskers correspond to outliers.

**Fig. 4:**
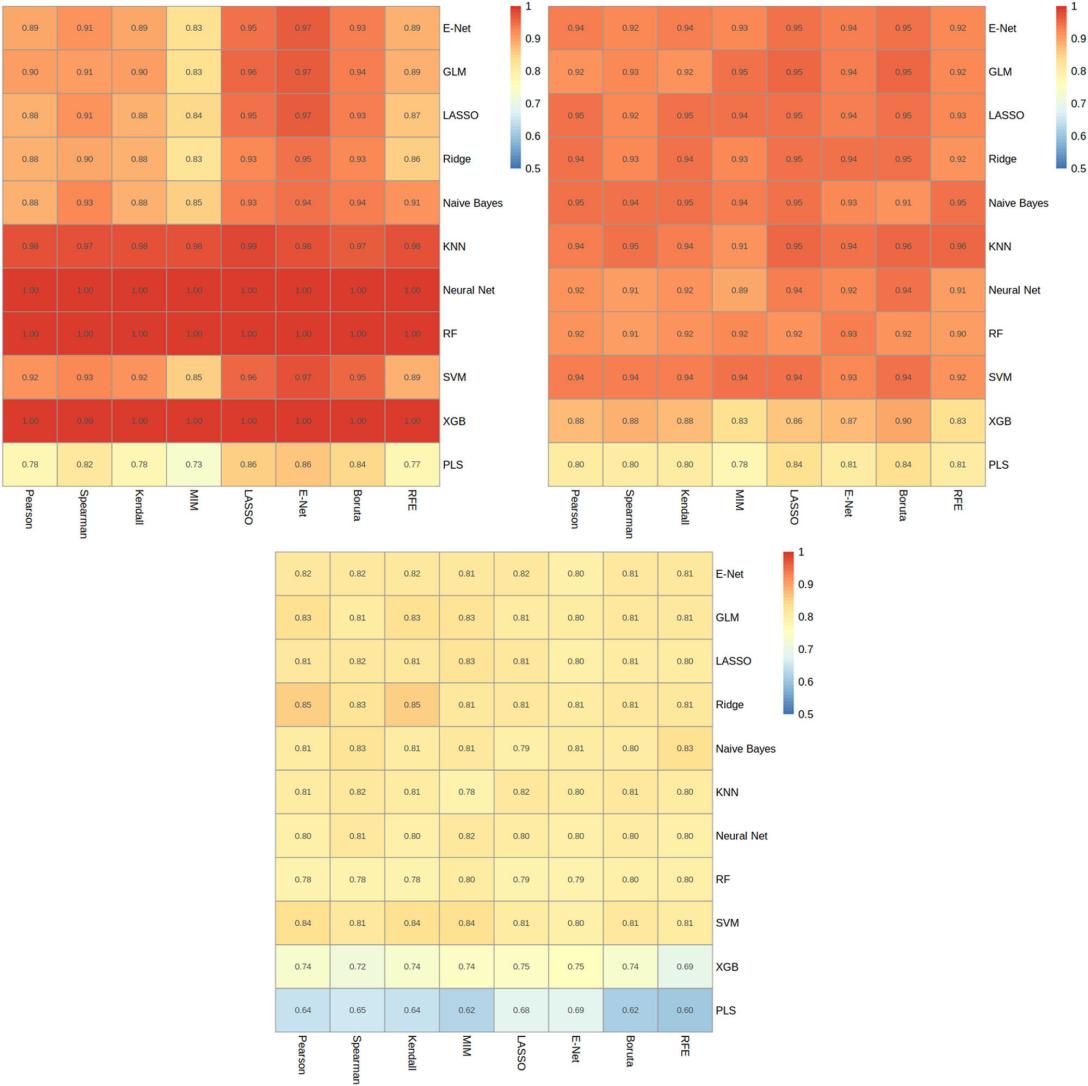
Supervised radiomics modelling. In heatmaps, x-axis corresponds to feature selection techniques and y-axis to modelling strategy; values are F1-scores. Plots (**A**)–(**C**), represent ICH training, ICH validation, and MPH test set results, respectively.

**Fig. 5:**
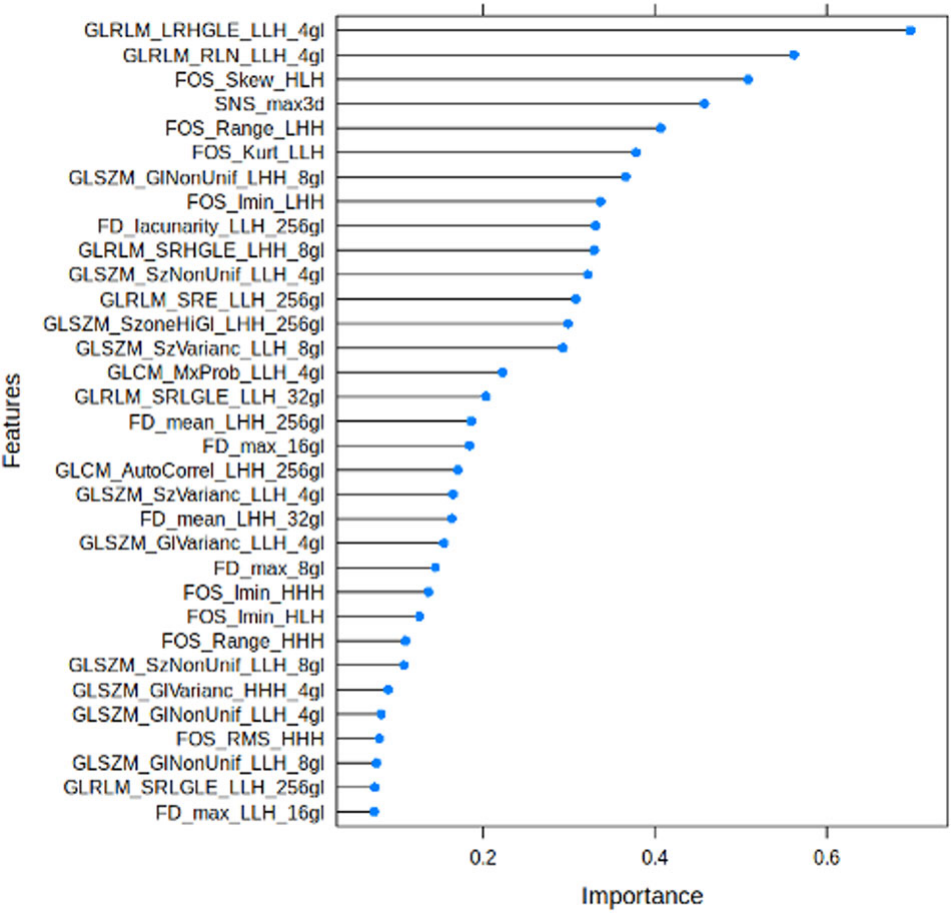
Feature Importance of Ridge Regression Model for the prediction of malignancy.

The best-performing segmentation model, utilised DL, achieved a dice surface coefficient (SD) of 0.85 ± 0.01, 0.88 ± 0.01 and 0.85 ± 0.01, for ICH training, ICH validation, and MPH test set, respectively (Figs. 1–3). The best-performing classification model, at a threshold of 0.5, termed the Ovarian Diagnostic Score (ODS), utilised ridge regressions with Pearson correlation-based feature reduction (Figs. 4, 5). The ODS model reached an F1-score of 0.88 (AUC 0.93) in the ICH training, 0.94 (AUC 0.89) in ICH validation and 0.83 (AUC 0.90) in the MPH external test set (Table 3).

**Table 3:** Performance of Ovarian Diagnostics score (ODS) radiomics model, Deep Learning (DL) models and CA-125 baseline model in the ICH Training, ICH validation, and MPH external test set

Model	ICH TRAINING					ICH VALIDATION					MPH TEST				
Metrics	F1	AUC	PREC	REC	SPEC	F1	AUC	PREC	REC	SPEC	F1	AUC	PREC	REC	SPEC
ODS	0.88	0.93	0.78	1.00	0.80	0.94	0.89	0.89	1.00	0.80	0.83	0.90	0.72	1.00	0.73
ResNet-18	0.93	0.97	0.93	0.93	0.95	0.81	0.85	0.85	0.77	0.79	0.75	0.80	0.82	0.78	0.83
ResNet-34	0.92	0.97	0.91	0.93	0.93	0.76	0.81	0.82	0.80	0.77	0.77	0.80	0.83	0.77	0.84
ResNet-50	0.88	0.96	0.91	0.84	0.93	0.37	0.61	0.41	0.34	0.35	0.33	0.54	0.40	0.29	0.28
CA-125	0.11	0.49	0.08	0.18	0.10	0.36	0.67	1.00	0.22	0.99	0.38	0.49	0.33	0.45	0.35

Performance metrics summarised: F1 score (F1), Area under the ROC curve (AUC), Precision (PREC), Recall (REC), and Specificity (SPEC).

The ODS model outperformed the CA-125-based model in the ICH validation (F1 score 0.94 vs. 0.36) and MPH test (F1 score 0.83 vs. 0.38), respectively. The ODS model had a recall of 1.00 in the ICH training, ICH validation and MPH test sets, respectively, so there were no false negative cases. The ODS model had a precision (PREC) of 0.78 in ICH training, 0.89 in ICH validation and 0.72 in the MPH test set and a specificity (SPEC) of 0.80 in both the ICH training and validation, and 0.73 in the MPH test set. The ODS model had a false discovery (FDR) and positive rate (FPR) of 28% and 27% in MPH, compared to 11% and 20%, respectively, in ICH validation.

The calibration curve for the ODS model had an intercept of 0.00000037, −0.87, −0.55 in the ICH training, ICH validation, MPH test set, respectively (a perfect intercept is 0) (Supplementary Fig. 1–3). The slope of the calibration curve evaluates the spread of the estimated risks (target value of 1). The model had a slope of 1.39, 1.06 and 0.25 in the ICH training, ICH validation and test set, respectively. The slope of the MPH test indicates that the estimated risks are too extreme, i.e. higher predicted probability for high-risk patients and lower predicted probability for low-risk patients, compared with the ICH validation dataset (with more moderate risk estimates). The over-prediction (false positive) pattern seen in the MPH test set, is reflected in the lower precision of 0.72, compared to 0.89 in the ICH validation (Supplementary Figs. 1–3).

## Discussion

We have demonstrated that the ODS model provides an end-to-end, method of adnexal mass segmentation and classification, with comparable predictive performance (F1 0.83, AUC 0.90) to the published performance of expert SA (AUC 0.96) and the ADNEX model (AUC 0.94). This ODS model has potential clinical utility, through its ability to automate the identification of the region of interest and provide a real-time classification of an adnexal mass, without the need for prior ultrasound operator experience.

The detection of malignancy is fundamental to any diagnostic test. Prioritising the detection of positive cases (recall/sensitivity) is often at the expense of specificity and can result in a high false discovery (FDR) and false positive rate (FPR). The ODS model had a recall (sensitivity) of 1.00 in the ICH validation and MPH test sets, translating to a high malignancy detection rate. The precision (positive predictive value) and specificity of the ODS model were 0.89 and 0.80 in the ICH validation and 0.72 and 0.73 in the MPH test set, corresponding to an FDR of 11 and 28% and an FPR of 20% and 27% in the ICH validation and MPH test set respectively. This is comparable to existing literature in the field, Li et al. (precision: 0.765 and 0.488, specificity: 0.797 and 0.843 in test sets 1 and 2, respectively). Given the high sensitivity of the ODS model, it could have value as an initial triage tool (first step), to identify ‘high risk’ adnexal masses, which warrant further evaluation by an expert ultrasound examiner (second step). This two-step triage approach would reduce the workload on an expert examiner, through focusing the review of ‘high risk’ cases and would also reduce the clinical impact of using an ODS model with a relatively high FDR and FPR. We would expect a further improvement in both the FDR and FPR, with expansion of the training dataset across multiple centres, to capture additional variance and improve the overall applicability of the ODS model. Furthermore, integration of other clinical metrics, including specific ultrasound features (solid components), has the potential to enhance the performance of the ODS model, but this is unfortunately beyond the remit of the focus of this study.

A direct comparison of the performance of the ODS model against existing literature in the field is limited by the lack of open-source code and image datasets. However, in the development of the ODS model, frequently used ML and DL-based approaches were evaluated. Using a radiomics-based approach coupled with a diverse training set may explain the superior predictive performance of the ODS model within the ICH validation (F1 score: 0.94) and MPH test set (F1-score: 0.83), compared to the two DL-based studies to date by Gao et al. (F1-score: 0.551) and Li et al. (F1-score: 0.684)^19,27^, suggesting that radiomics-based approaches may be more suited to smaller clinical datasets. The drop in the performance of the ODS model between our internal and external test sets, is expected given the heterogeneity of adnexal mass sub-types (*p* < 0.01), malignancy rates (*p* < 0.05), scanner types and geographically diverse locations between the validation and test datasets. Furthermore, previous studies^18,21,31^ have used AUC as the performance metric, rather than F1-score, which may have overestimated the respective model’s performance at the classification of adnexal masses, given the lack of adjustment for class imbalance. Several ML models published in the literature have not been evaluated in an external test set; thus, limiting the conclusions that can be drawn on their performance and overall generalisability.

This is the largest radiomics study of ultrasound classification of adnexal masses to date, incorporating 577 cases (1444 images) in the development dataset (ICH) and externally validated (MPH) on 184 test cases (476 images). To facilitate the application of the ODS model to all adnexal masses (including those selected for conservative management), the training set included expectantly and surgically (histology) managed adnexal masses (Table 1). A two-step segmentation pipeline was implemented, involving an expert review of each adnexal mass image to ensure the dataset is of the highest quality, whilst minimising operator bias.

The methodical model development pipeline involved the evaluation of eight feature reduction and 11 ML techniques in various combinations, to establish the best performing ML model (ODS model) for this classification task. The ODS model at a threshold of 0.5 performed well in both the ICH (validation, F1 0.94, AUC 0.89) and MPH (external, F1 0.83, AUC 0.90) test sets, demonstrating its generalisability and applicability. In addition to the radiomics models, we also developed ResNet based DL models for the classification of malignancies without the need for manual segmentation. Whilst ResNet based architectures performed well in training, there was a decrease in performance in the validation and test sets, which was also seen in Gao et al.‘s paper^19^. This is a key indicator of model overfitting especially with the largest ResNet-50 architecture despite our augmentation efforts. Overfitting is a common occurrence in DL models; however, it is expected that with an increase in data set sizes this issue would be addressed.

Our results demonstrate the potential benefit of an end-to-end model capable of triaging adnexal masses. The main limitation of this study is its sample size, which when coupled with significant adnexal mass heterogeneity can make a ML model prone to overfitting. This is demonstrated in the observed drop in classification performance and calibration of the ODS model between the ICH Validation and MPH test set. Establishing a large multi-centre cohort will look to overcome the challenges associated with relatively small datasets and aim to improve the overall calibration of radiomics (ML) and DL models and their potential clinical translation. Secondly, the retrospective extraction of adnexal mass images for the development of the ML model restricted the prospective application of ADNEX and RMI models to enable direct comparison against existing adnexal mass classification approaches. The performance of existing clinical models within the literature (RMI, SR and ADNEX) is evaluated using AUC. The performance of the ODS model on an external test set using AUC was 0.90 (F1 score 0.83), which is comparable to RMI (AUC 0.89), but slightly inferior to expert SA (AUC 0.96) and ADNEX (AUC 0.94), respectively^11,12^. Whilst the performance of the ML model has not surpassed the published performance of existing classification models, the ODS model does offer a potential end-to-end diagnostic approach, that does not require expertise in adnexal mass classification. We aim to prospectively validate the ODS model, against existing adnexal mass classification methods (SA, RMI, ADNEX and ORADS-US classification system) to establish its value as a potential diagnostic tool. Furthermore, evaluation across multiple centres, in the hands of operators of varying experience is important, to establish the generalisability and clinical utility of this ODS model.

To contextualise the ODS model’s performance, deep learning and radiomics have shown similar success in other pathologies, achieving AUCs of 0.90^32^, 0.72^33^, 0.83^34^, 0.76^35^ and 0.72^36^ in differentiating lung metastases, predicting lymph node metastasis in breast cancer, classifying lymph nodes in lung cancer, predicting human epidermal growth factor receptor 2 (HER2) status in breast cancer and improving lung cancer diagnosis, respectively. The ODS model’s performance (F1-score 0.83, AUC 0.90) is aligned with these applications, underscoring its potential in adnexal mass classification.

A radiomics-based model offers a degree of explainability of tissue biophysics to the classification output. The radiomics features Grey Level Run Length Matrix (GLRLM) and a Grey Level Size Zone (GLSZM) were thought to be of high importance in the malignant class, therefore dominated the feature space that defined ODS i.e. to detect the presence of malignancy. GLRLM quantifies grey level runs that are the length in number of pixels, of consecutive pixels that have the same grey level value while GLSZM quantifies grey level zones that are the number of connected pixels that share the same grey level intensity, so reflect the heterogeneity seen within malignant masses and biophysically begins to define the distinguishing features of benign versus malignant adnexal masses.

We have developed a high-performing automated end-to-end ML model, capable of accurately classifying adnexal masses, with a good malignancy detection rate. Subject to further external validation, the ML model may be widely applicable, given the consistent performance of the ODS model across both internal and external datasets. An ML model could offer a scalable, accurate triage tool to effectively identify cases deemed at ‘high risk’ of malignancy, which would warrant further expert ultrasound evaluation. A model, which is not reliant upon ultrasound expertise to classify adnexal masses, could address inherent barriers limiting the use of existing ultrasound-based models. Further work is required to evaluate the performance of the ODS model prospectively against existing methods of adnexal mass classification (expert SA, RMI, SR, ORAD-US and ADNEX) to further establish its role within the adnexal mass classification pipeline.

## Methods

### Study design and participating cohorts

This retrospective study consisted of women (≥18 years) recruited from two European Gynaecology Oncology centres, between December 2017 and September 2022: (1) Imperial College Healthcare NHS Trust, London, UK (ICH) and (2) Morgagni-Pierantoni Hospital, Forli, Italy (MPH). The ML model was developed and internally validated on adnexal mass images from ICH (Development *n* = 577 masses; 1444 images) and externally validated utilising the MPH dataset (test *n* = 184 masses; 476 images).

Eligibility criteria included: a non-physiological adnexal mass which had been: (1) expectantly managed for 6 months and classified as benign according to ultrasound expert SA or (2) undergone surgical removal with available histology. Exclusion criteria included: only transabdominal ultrasound images, physiological cysts, and cases with only ‘split screen’ images. Pregnancy was not an exclusion criterion.

### Ultrasound image acquisition

All ultrasound examinations were carried out using a standardised approach, with the application of IOTA terms and definitions^37^. Expert subjective assessment (SA) was used to classify the adnexal mass(es) as benign or malignant. Adnexal masses were scanned on various ultrasound systems including: Voluson GE (GE Healthcare, Zipf, Austria: Voluson E6, E8, E9, E10, P8, S10), Samsung (Samsung Medison, Seoul, Republic of Korea: W10, WS80, HS60) and Esaote (Esaote S.p.A, Genoa, Italy: MyLab). 2D transvaginal ultrasound, grayscale adnexal mass images were taken for each adnexal mass. A proportion of ultrasound images 150 images (31.5%) in MPH dataset had callipers present. The ultrasound examinations were carried out by ultrasound examiners of varying experience, but under expert supervision. The pseudo-anonymised grayscale adnexal mass images were exported as TIFF images. Adnexal masses which required surgery because of symptoms, suspicion of malignancy or patient choice were removed by an appropriately trained surgeon within the centres. The World Health Organisation (WHO) classification was used to define the tumour sub-type and the International Federation of Gynaecology and Obstetrics criteria was used to stage malignant adnexal masses^38^. Clinical data, including age and CA-125 level was also collected where available.

### Ethical approval

This study was approved by the UK Regional Ethics Committee (05/QO406/178). All procedures involving human participants were in accordance with the ethical standards of the institutional and/or national research committee and with the principles of the 1964 Declaration of Helsinki and its later amendments or comparable ethical standards. The use of anonymous external dataset images was granted by Imperial College Ethics Committee (22IC7780), all participants provided written informed consent.

### Model development

The region of interest was defined in a process known as segmentation within 3D Slicer (https://www.slicer.org/), an open-source segmentation software by an experienced ultrasound operator (J.F.B.)^39^. The segmented images were checked individually by a level III (expert) ultrasound examiner (C.L, M.M, S.S, N.B)^40^. In accordance with the Image Biomarkers Standardization Initiative (IBSI) scans were resampled to isotropic 1 × 1 mm^2 ^^41^. All continuous variables were scaled and mean-centred using training data set statistics.

To enable the development of an end-to-end classification model, the first step involves the development of a segmentation model, to identify the region of interest (lesion), the second step requires the development of a classification model to determine if the lesion is benign or malignant.

We developed several convolutional neural network (CNN)-based segmentation models, through training on the ICH development training dataset (462 masses, 1155 images). The segmentation performance was evaluated on (1) ICH validation (115 masses 289 images) and (2) MPH test set (184 masses, 476 images).

Radiomics features were extracted using TexLab 2.0, with various grey-level binning ranges, to compute a total of 3906 radiomics-based features. We explored a combination of 8 different feature reduction techniques with 11 different ML algorithms, including linear and tree-based techniques, and boosted and regularised variations. Feature reduction methods included mutual information, recursive feature elimination, correlation-based and linear methods. Hyper-parameter optimisation was performed via grid-search with ten-fold cross-validation optimising for concordance index. Hyper-parameter ranges of the models are listed in Supplementary Table 4. The binarization of predicted probabilities (malignant vs benign) was derived from a threshold of 0.5.

In addition to traditional radiomics features, we also built several CNN classification models. ResNet-based DL architectures were applied without the segmentation region of interest (ResNet-18,-32, and -50).

We used the Radiomics Quality Score^42^ and Transparent Reporting of a Multi-variable Prediction Model for Individual Prognosis or Diagnosis (TRIPOD, https://www.tripod-statement.org/) guidelines for reporting the development and validation of the prediction models (Supplementary Tables 1 and 2).

### Outcomes

The primary outcome of the segmentation ML model was the identification of the region of interest, compared to ultrasound examiners segmentation (ground truth). The primary outcome of the classification ML model was adnexal mass diagnosis (benign or malignant), compared to ultrasound SA or histological diagnosis.

### Statistical analysis

To assess the model’s performance at the accurate classification of an adnexal mass, we used the F1-Score (harmonic mean of precision and recall), precision and recall (sensitivity). For clinical relevance we also calculated Area under the ROC curve (AUC), and specificity. We assessed the calibration of the ODS model, through evaluation of the calibration intercept and slope for ICH training, ICH validation and MPH test set (Supplementary Figs. 1–3). The purpose of calibration was to determine whether the ODS model over or underpredicted the risk. Quantitative statistics were presented as median and interquartile range (IQR). Continuous variables were compared using Wilcoxon signed-rank tests, and categorical variables were compared using the Fisher’s exact test. Statistical analysis of clinical variables was two-sided, and Benjamini–Hochberg multiple testing corrected *p* values of less than 0.05 were used to indicate statistical significance. Calibration curve statistics were computed using the Regression Modelling Strategies package version 6.5, using the non-parametric confidence intervals method described by Qin and Hotilovac^43^. With the single variable CA-125 model, we performed a univariate logistic regression. Missing CA-125 values were imputed using the multivariate imputation by chained equations algorithm.

### Feature selection

In this study, conducted entirely in R, a variety of feature selection techniques were integrated to enhance the robustness and interpretability of our machine learning models. Regularisation methods, such as LASSO and Elastic Net regression, were utilised via the glmnet package^44^. These methods apply penalties to the coefficients of a linear model, thereby reducing overfitting and leading to the selection of a subset of key features. LASSO employs an L1 penalty to force some coefficients to zero, while Elastic Net combines both L1 and L2 penalties, incorporating features of both ridge regression and LASSO.

Correlation-based methods including Pearson, Spearman’s, and Kendall’s rank correlation were also employed. Implemented using the ‘corr’ package in R, these methods select features based on their individual characteristics, with Pearson assuming linear relationships and Spearman’s and Kendall’s focusing on monotonic relationships.

Univariate logistic regression was another approach employed, using the glm package in R^45^. This technique involved assessing each feature with the outcome variable and selecting those with a *p* value less than 0.05, adjusted for multiple comparisons using the Benjamini & Hochberg method^46^.

Recursive Feature Elimination (RFE), implemented with the ‘rfeControl’ package in R and utilising a random forest model^47^, was used to iteratively eliminate features based on their importance, employing cross-validation to identify the most effective subset.

Mutual Information^48^, was incorporated to evaluate the relevance and redundancy of features in predicting our target variable. The Boruta method^49^ was employed as a wrapper around a Random Forest classifier for iteratively removing less relevant features through statistical testing.

### Machine learning algorithms

Our classification models, all developed in R, utilised a diverse array of supervised algorithms. Logistic Regression (LR)^50^, along with its regularised variations LASSO and Elastic Net, were key components in our modelling approach. Linear Support Vector Machines (L-SVM)^51^ were also employed for their ability to classify data by finding an optimal separating hyperplane. The K-Nearest Neighbors (KNN) algorithm^52^ was used for its simplicity and effectiveness, classifying data based on the proximity of points in the feature space. Ensemble decision-tree-based models like Random Forest (RF) and Extreme Gradient Boosting Machines (XGB)^53,54^ were selected for their robustness and accuracy in handling complex classification tasks. Partial Least Squares (PLS)^55^ was particularly useful in scenarios where we dealt with a large number of features and significant collinearity among them. For certain classification tasks, a Single-Layer Feed-Forward Neural Network (NNET)^56^, implemented using the ‘nnet’ package, was found to be effective. In addition, the Naïve-Bayes (NB) algorithm^57^was employed for its probabilistic approach to classification, leveraging Bayes’ theorem and assuming conditional independence among features.

### Reporting summary

Further information on research design is available in the Nature Research Reporting Summary linked to this article.

### Supplementary information

Supplementary Material

REPORTING SUMMARY

## Data Availability

The anonymized adnexal image datasets and corresponding clinical metadata used for model development and validation in this study are not publicly available due to privacy and ethical considerations. However, these datasets can be made accessible to qualified researchers upon reasonable request, any specific accession codes or unique identifiers associated with the datasets will be provided upon approval of the request.

## Code availability

Scripts for machine learning are indicated in the methods section. The R and Python scripts used to reproduce the key findings and generate figures have been made available alongside the deep learning segmentation and classification models code at https://github.com/Cancer-Imaging-Group/adnexal-mass-classifier.
