# Supplementary material for: Machine learning and radiomics for segmentation and classification of adnexal masses on ultrasound
Source: NPJ Precis Oncol. 2024 Feb 20;8:41. doi: 10.1038/s41698-024-00527-8 (PMC10879532; doi:10.1038/s41698-024-00527-8)
Supplement: Supplementary file 1 — Supplementary Material [file 41698_2024_527_MOESM1_ESM.pdf]

Supplementary Table 1: The Radiomics Quality Score for this study is summarised below. Each criterion is evaluated for points, and our scores are detailed accordingly.

Criteria	Points	Our Score
1. Image protocol quality -– well-documented image protocols (for example, contrast, slice thickness, energy, etc.) and/or usage of public image protocols allow reproducibility/replicability	+ 1 (if protocols are well-documented) + 1 (if public protocol is used)	0
2. Multiple segmentations -– possible actions are: segmentation by different physicians/algorithms/software, perturbing segmentations by (random) noise, segmentation at different breathing cycles. Analyse feature robustness to segmentation variabilities	+ 1	0
3. Phantom study on all scanners -– detect inter-scanner differences and vendor-dependent features. Analyse feature robustness to these sources of variability	+ 1	0
4. Imaging at multiple time points -– collect images of individuals at additional time points. Analyse feature robustness to temporal variabilities (for example, organ movement, organ expansion/shrinkage)	+ 1	0
5. Feature reduction or adjustment for multiple testing -– decreases the risk of overfitting. Overfitting is inevitable if the number of features exceeds the number of samples. Consider feature robustness when selecting features	– 3 (if neither measure is implemented) + 3 (if either measure is implemented)	3
6. Multivariable analysis with non-radiomics features (for example, EGFR mutation) -– is expected to provide a more	+ 1	1

holistic model. Permits correlating/inferencing between radiomics and non-radiomics features		
7. Detect and discuss biological correlates -- demonstration of phenotypic differences (possibly associated with underlying gene–protein expression patterns) deepens understanding of radiomics and biology	+ 1	1
8. Cut-off analyses -- determine risk groups by either the median, a previously published cut-off or report a continuous risk variable. Reduces the risk of reporting overly optimistic results	+ 1	1
9. Discrimination statistics -- report discrimination statistics (for example, C-statistic, ROC curve, AUC) and their statistical significance (for example, p-values, confidence intervals). One can also apply resampling method (for example, bootstrapping, cross-validation)	+ 1 (if a discrimination statistic and its statistical significance are reported) + 1 (if a resampling method technique is also applied)	2
10. Calibration statistics -- report calibration statistics (for example, Calibration-in-the-large/slope, calibration plots) and their statistical significance (for example, *P*-values, confidence intervals). One can also apply resampling method (for example, bootstrapping, cross-validation)	+ 1 (if a calibration statistic and its statistical significance are reported) + 1 (if a resampling method technique is also applied)	2
11. Prospective study registered in a trial database -- provides the highest level of evidence supporting the clinical validity and usefulness of the radiomics biomarker	+ 7 (for prospective validation of a radiomics signature in an appropriate trial)	0
12. Validation -- the validation is performed without retraining and without adaptation of the cut-off value, provides crucial information with regard to credible clinical performance	- 5 (if validation is missing) + 2 (if validation is based on a dataset from the same institute) + 3 (if validation is based on a dataset from	4

	another institute) + 4 (if validation is based on two datasets from two distinct institutes) + 4 (if the study validates a previously published signature) + 5 (if validation is based on three or more datasets from distinct institutes)<br>*Datasets should be of comparable size and should have at least 10 events per model feature	
13. Comparison to "gold standard" -– assess the extent to which the model agrees with/is superior to the current "gold standard" method (for example, TNM-staging for survival prediction). This comparison shows the added value of radiomics	+ 2	2
14. Potential clinical utility -– report on the current and potential application of the model in a clinical setting (for example, decision curve analysis).	+ 2	2
15. Cost-effectiveness analysis - report on the cost-effectiveness of the clinical application (for example, QALYs generated)	+ 1	0
16. Open science and data - make code and data publicly available. Open science facilitates knowledge transfer and reproducibility of the study	+ 1 (if scans are open source) + 1 (if region of interest segmentations are open source) + 1 (if code is open source) + 1 (if radiomics features are calculated on a set of representative ROIs and the calculated features and	0

	representative ROIs are open source)	
Total points (36 = 100%)	36	18 (50%)

Supplementary table 2: TRIPOD recommendations, indicating the where the checklist items are located in the paper.

Section/Topic		Checklist Item	Page
**The Use of Machine Learning Models and Radiomics for Segmentation and Classification of Adnexal Masses on Ultrasound: A multi-cohort retrospective study**			
Title	1	Identify the study as developing and/or validating a multivariable prediction model, the target population, and the outcome to be predicted.	1
Abstract	2	Provide a summary of objectives, study design, setting, participants, sample size, predictors, outcome, statistical analysis, results, and conclusions.	2
**Introduction**			
Background and objectives	3a	Explain the medical context (including whether diagnostic or prognostic) and rationale for developing or validating the multivariable prediction model, including references to existing models.	3-5
	3b	Specify the objectives, including whether the study describes the development or validation of the model or both.	5
**Methods**			
Source of data	4a	Describe the study design or source of data (e.g., randomized trial, cohort, or registry data), separately for the development and validation data sets, if applicable.	12
	4b	Specify the key study dates, including start of accrual; end of accrual; and, if applicable, end of follow-up.	12
Participants	5a	Specify key elements of the study setting (e.g., primary care, secondary care, general population) including number and location of centres.	12
	5b	Describe eligibility criteria for participants.	12

	5c	Give details of treatments received, if relevant.	N/A
Outcome	6a	Clearly define the outcome that is predicted by the prediction model, including how and when assessed.	12
	6b	Report any actions to blind assessment of the outcome to be predicted.	N/A
Predictors	7a	Clearly define all predictors used in developing or validating the multivariable prediction model, including how and when they were measured.	13-14
	7b	Report any actions to blind assessment of predictors for the outcome and other predictors.	N/A
Sample size	8	Explain how the study size was arrived at.	N/A
Missing data	9	Describe how missing data were handled (e.g., complete-case analysis, single imputation, multiple imputation) with details of any imputation method.	15
Statistical analysis methods	10a	Describe how predictors were handled in the analyses.	13-15
	10b	Specify type of model, all model-building procedures (including any predictor selection), and method for internal validation.	13-15
	10d	Specify all measures used to assess model performance and, if relevant, to compare multiple models.	13-15
Risk groups	11	Provide details on how risk groups were created, if done.	N/A
**Results**			
Participants	13a	Describe the flow of participants through the study, including the number of participants with and without the outcome and, if applicable, a summary of the follow-up time. A diagram may be helpful.	6, Table 1
	13b	Describe the characteristics of the participants (basic demographics, clinical features, available predictors), including the number of participants with missing data for predictors and outcome.	Table 1
Model development	14a	Specify the number of participants and outcome events in each analysis.	Table 1
	14b	If done, report the unadjusted association between each candidate predictor and outcome.	NA

Model specification	15a	Present the full prediction model to allow predictions for individuals (i.e., all regression coefficients, and model intercept or baseline survival at a given time point).	SM, 16-17
	15b	Explain how to the use the prediction model.	13-17 + GitHUB
Model performance	16	Report performance measures (with CIs) for the prediction model.	Table 3
**Discussion**			
Limitations	18	Discuss any limitations of the study (such as nonrepresentative sample, few events per predictor, missing data).	10
Interpretation	19b	Give an overall interpretation of the results, considering objectives, limitations, and results from similar studies, and other relevant evidence.	8-10
Implications	20	Discuss the potential clinical use of the model and implications for future research.	8
**Other information**			
Supplementary information	21	Provide information about the availability of supplementary resources, such as study protocol, Web calculator, and data sets.	17
Funding	22	Give the source of funding and the role of the funders for the present study.	17-18
SM = Supplementary Material			

Supplementary Table 3: Radiomics Features of ODS Model. Feature represents the radiomics feature computed and the description briefly defines what this feature means.

Feature	Description
**SNS_max3d**	Maximum 3D diameter of the region of interest
**GLSZM_SzNonUnif_8gl**	Size-zone non-uniformity for a grey-level quantization of 8
**FD_max_8gl**	Maximum fractal dimension for a grey-level quantization of 8
**FD_max_16gl**	Maximum fractal dimension for a grey-level quantization of 16
**FOS_Kurt_LLH**	Kurtosis of the first-order statistics in the low-low-high direction
**GLSZM_GlNonUnif_LLH_4gl**	Grey-level non-uniformity in the low-low-high direction for a grey-level quantization of 4
**GLSZM_SzNonUnif_LLH_4gl**	Size-zone non-uniformity in the low-low-high direction for a grey-level quantization of 4
**GLSZM_GlVarianc_LLH_4gl**	Grey-level variance in the low-low-high direction for a grey-level quantization of 4
**GLSZM_SzVarianc_LLH_4gl**	Size-zone variance in the low-low-high direction for a grey-level quantization of 4
**GLRLM_RLN_LLH_4gl**	Run length non-uniformity in the low-low-high direction for a grey-level quantization of 4
**GLRLM_LRHGLE_LLH_4gl**	High grey-level run emphasis in the low-low-high direction for a grey-level quantization of 4
**GLCM_MxProb_LLH_4gl**	Maximum probability in the low-low-high direction for a grey-level quantization of 4
**GLSZM_GlNonUnif_LLH_8gl**	Grey-level non-uniformity in the low-low-high direction for a grey-level quantization of 8
**GLSZM_SzNonUnif_LLH_8gl**	Size-zone non-uniformity in the low-low-high direction for a grey-level quantization of 8
**GLSZM_GlVarianc_LLH_8gl**	Grey-level variance in the low-low-high direction for a grey-level quantization of 8
**GLSZM_SzVarianc_LLH_8gl**	Size-zone variance in the low-low-high direction for a grey-level quantization of 8
**FD_max_LLH_16gl**	Maximum fractal dimension in the low-low-high direction for a grey-level quantization of 16
**GLRLM_SRLGLE_LLH_32gl**	Short run low grey-level emphasis in the low-low-high direction for a grey-level quantization of 32
**GLSZM_GlNonUnif_LLH_128gl**	Grey-level non-uniformity in the low-low-high direction for a grey-level quantization of 128
**GLRLM_SRE_LLH_256gl**	Short run emphasis in the low-low-high direction for a grey-level quantization of 256
**GLRLM_SRLGLE_LLH_256gl**	Short run low grey-level emphasis in the low-low-high direction for a grey-level quantization of 256
**FD_lacunarity_LLH_256gl**	Fractal dimension lacunarity in the low-low-high direction for a grey-level quantization of 256
**FOS_Imin_LHH**	Minimum intensity in the low-high-high direction
**FOS_Range_LHH**	Intensity range in the low-high-high direction
**GLSZM_GlNonUnif_LHH_8gl**	Grey-level non-uniformity in the low-high-high direction for a grey-level quantization of 8
**GLSZM_SzVarianc_LHH_8gl**	Size-zone variance in the low-high-high direction for a grey-level quantization of 8
**GLRLM_SRHGLE_LHH_8gl**	Short run high grey-level emphasis in the low-high-high direction for a grey-level quantization of 8
**FD_mean_LHH_32gl**	Mean fractal dimension in the low-high-high direction for a grey-level quantization of 32

**GLSZM_SzoneHiGl_LHH_256gl**	Small zone high grey-level emphasis in the low-high-high direction for a grey-level quantization of 256
**FD_mean_LHH_256gl**	Mean fractal dimension in the low-high-high direction for a grey-level quantization of 256
**GLCM_InfCo2_LHH_256gl**	Informational measure of correlation 2 in the low-high-high direction for a grey-level quantization of 256
**GLCM_AutoCorrel_LHH_256gl**	Autocorrelation in the low-high-high direction for a grey-level quantization of 256
**FOS_Imin_HLH**	Minimum intensity in the high-low-high direction
**FOS_Range_HLH**	Intensity range in the high-low-high direction
**FOS_Skew_HLH**	Skewness in the high-low-high direction
**FOS_RMS_HLH**	Root mean square in the high-low-high direction
**FOS_Imin_HHH**	Minimum intensity in the high-high-high direction
**FOS_Range_HHH**	Intensity range in the high-high-high direction
**FOS_RMS_HHH**	Root mean square in the high-high-high direction
**GLSZM_GlVarianc_HHH_4gl**	Grey-level variance in the high-high-high direction for a grey-level quantization of 4

Supplementary Table 4: Overview of Hyperparameters for Various Machine Learning Models. This table presents a detailed summary of key hyperparameters for a range of machine learning models, including Elastic Net, Generalized Linear Model, LASSO, Ridge Regression, Naive Bayes, K-Nearest Neighbors, Neural Network, Random Forest, Support Vector Machine, Extreme Gradient Boosting, and Partial Least Squares. It includes the grid search values and brief descriptions to aid in understanding and tuning these models for optimal performance.

**Model**	**Library**	**Hyperparameter**	**Grid Search Values**	**Description**
E-Net	glmnet	Alpha	0 to 1 (several steps)	Mixing parameter for Elastic Net Regression
E-Net	glmnet	Lambda	Logarithmically spaced	Regularization strength
GLM	glmnet	Lambda	Logarithmically spaced	Regularization strength for Generalized Linear Model
LASSO	glmnet	Lambda	Logarithmically spaced	Regularization strength for LASSO Regression
Ridge	glmnet	Lambda	Logarithmically spaced	Regularization strength for Ridge Regression
Naive Bayes	naivebayes	Laplace	0, 1, etc.	Smoothing parameter
Naive Bayes	naivebayes	usekernel	TRUE/FALSE	Whether to use kernel density estimates
KNN	caret	k	5, 7, 9, etc.	Number of neighbors
Neural Net	nnet	size	Varies (e.g., 1, 5, 10)	Number of units in the hidden layer
Neural Net	nnet	decay	Varies (e.g., 0.1, 0.01)	Weight decay for regularization
RF	randomForest	ntree	500, 1000, 1500, etc.	Number of trees in the forest
RF	randomForest	mtry	1 to number of variables	Number of variables randomly sampled as candidates
SVM	kernlab	sigma	Varies widely	Kernel width parameter
SVM	kernlab	C	0.1, 1, 10, etc.	Regularization parameter
XGB	xgboost	nrounds	100, 200, 300, etc.	Number of boosting rounds

XGB	xgboost	eta (learning rate)	0.01, 0.05, 0.1, 0.3	Step size shrinkage used in update
XGB	xgboost	max_depth	3, 4, 5, 6, 10	Maximum depth of a tree
XGB	xgboost	subsample	0.5, 0.7, 1	Subsample ratio of the training instances
XGB	xgboost	colsample_bytree	0.5, 0.7, 1	Subsample ratio of columns when constructing a tree
PLS	pls	ncomp	1, 2, 3, ...	Number of components to use in the model

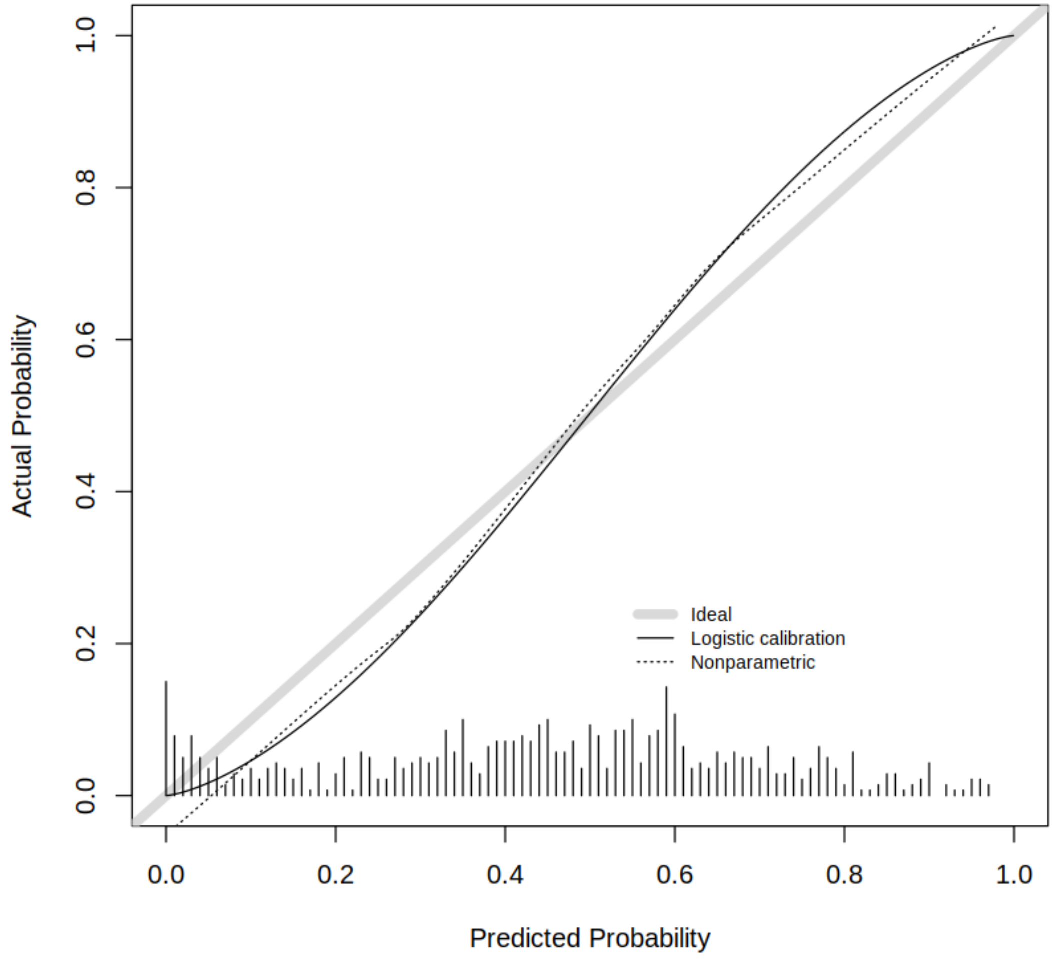

Supplementary Figure 1: Calibration Curve ICH Training Data. Logistic Calibration and Nonparametric calibration curves indicated for the ODS model. Slope=1.39 (95% CI:1.126-1.62), intercept=0.00000037(95% CI: 0.18-0.18)

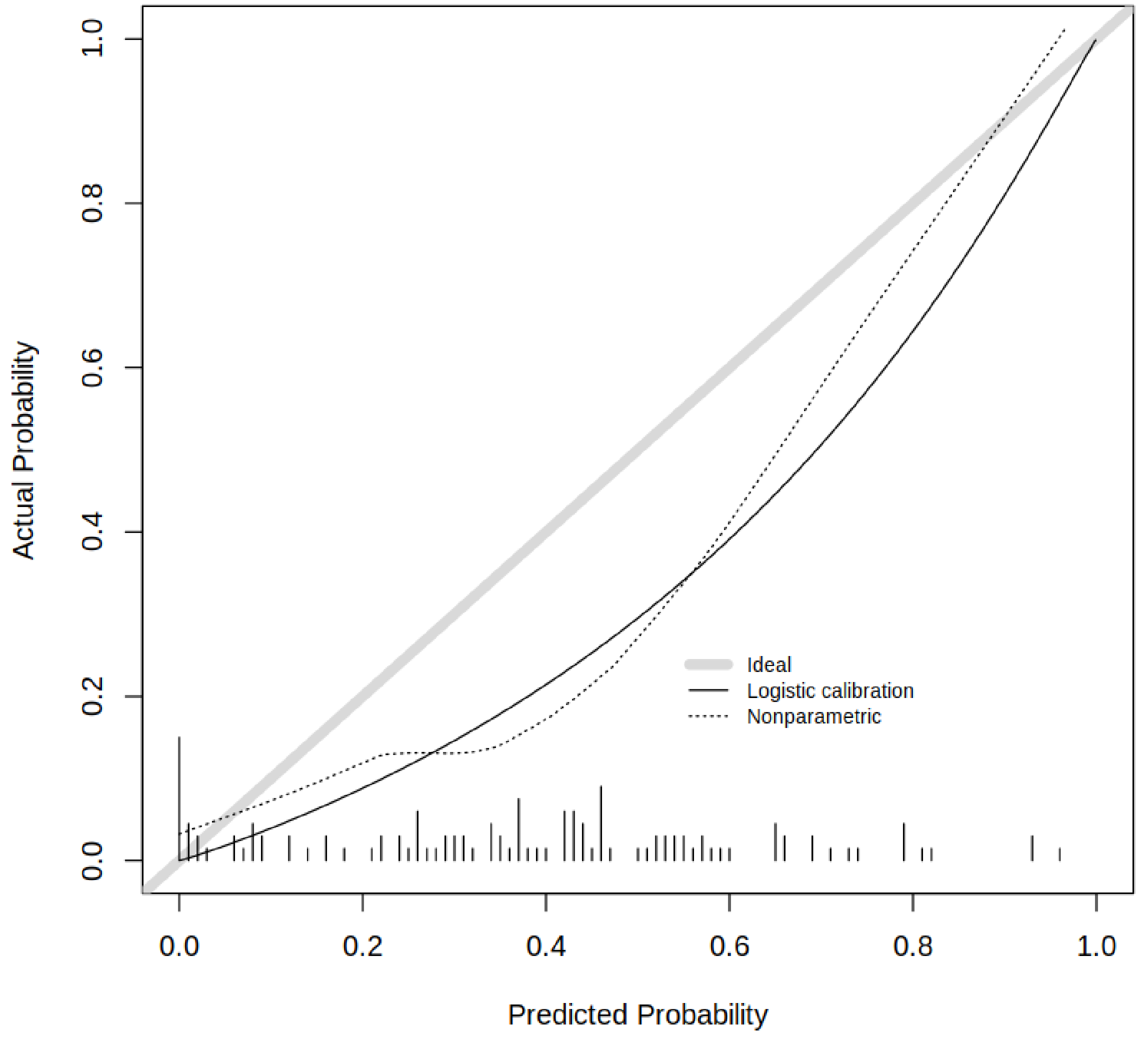

Supplementary Figure 2: Calibration Curve ICH Validation Data. Logistic Calibration and Nonparametric calibration curves indicated for the ODS model. Slope=1.06 (95% CI: 0.51 – 1.61), Intercept= -0.87 (95% CI: -1.35 -0.38)

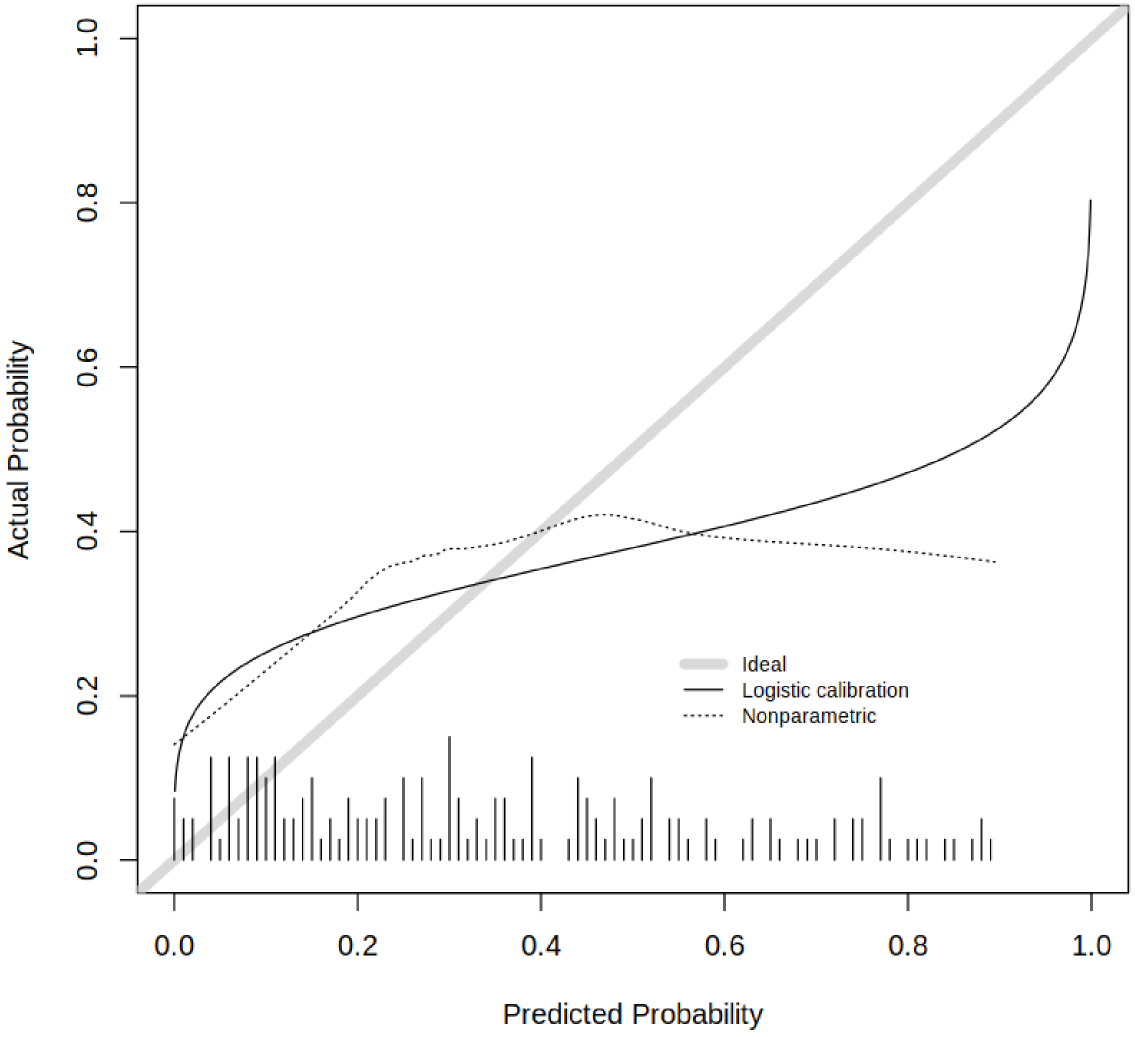

Supplementary Figure 3: Calibration Curve MPH Test Data. Logistic Calibration and Nonparametric calibration curves indicated for the ODS model. Slope=0.25 (95% CI: 0.06 – 0.44), intercept= -0.55 (95% CI: --0.91 - -0.18).

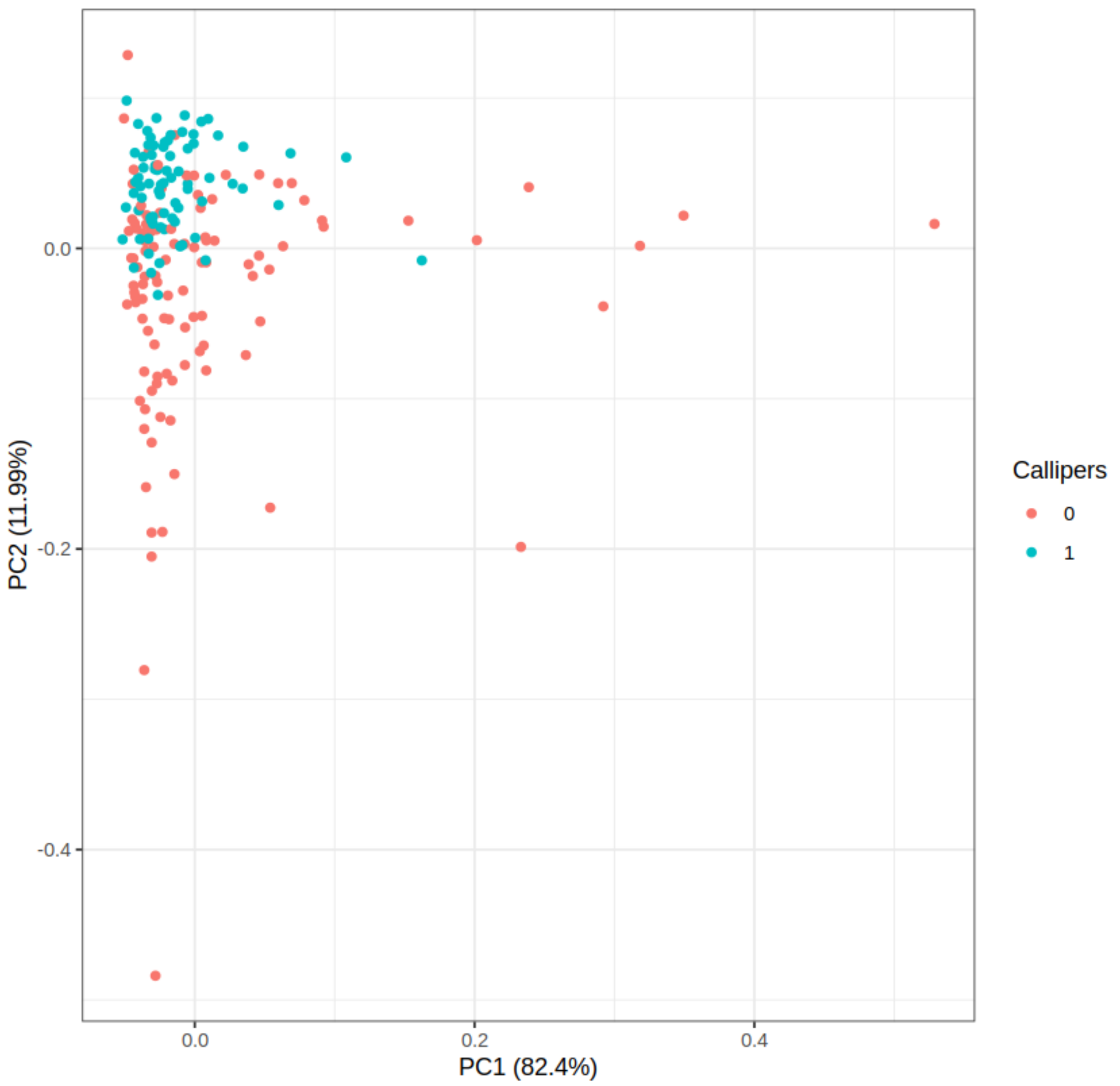

Supplementary Figure 4: Principal Component Analysis of Radiomics Features of the Test Data. PCA components derived from features used in the ODS model. First two principal components plotted on the X and Y axis respectively. Colours correspond to if callipers were present in the original ultrasound image.
